# Prediction of apoptosis protein subcellular location based on amphiphilic pseudo amino acid composition

**DOI:** 10.3389/fgene.2023.1157021

**Published:** 2023-02-28

**Authors:** Wenxia Su, Shuyi Deng, Zhifeng Gu, Keli Yang, Hui Ding, Hui Chen, Zhaoyue Zhang

**Affiliations:** ^1^ College of Science, Inner Mongolia Agriculture University, Hohhot, China; ^2^ School of Life Science and Technology, Center for Information Biology, University of Electronic Science and Technology of China, Chengdu, China; ^3^ Nonlinear Research Institute, Baoji University of Arts and Sciences, Baoji, China; ^4^ School of Healthcare Technology, Chengdu Neusoft University, Chengdu, China

**Keywords:** apoptosis protein, subcellular location, amphiphilic pseudo amino acid composition, support vector machine, jackknife test

## Abstract

**Introduction:** Apoptosis proteins play an important role in the process of cell apoptosis, which makes the rate of cell proliferation and death reach a relative balance. The function of apoptosis protein is closely related to its subcellular location, it is of great significance to study the subcellular locations of apoptosis proteins. Many efforts in bioinformatics research have been aimed at predicting their subcellular location. However, the subcellular localization of apoptotic proteins needs to be carefully studied.

**Methods:** In this paper, based on amphiphilic pseudo amino acid composition and support vector machine algorithm, a new method was proposed for the prediction of apoptosis proteins\x{2019} subcellular location.

**Results and Discussion:** The method achieved good performance on three data sets. The Jackknife test accuracy of the three data sets reached 90.5%, 93.9% and 84.0%, respectively. Compared with previous methods, the prediction accuracies of APACC_SVM were improved.

## 1 Introduction

Apoptosis is a type of programmed cell death mechanism that eliminates unnecessary or damaged cells from the body for cellular homeostasis regulation. The apoptotic program is executed by multiple pathways and controlled by the interactions between several molecules. Apoptosis proteins, such as the inhibitor of apoptosis protein (IAP) family, are proteins involved in the process of cell apoptosis for various stress responses. The different functions of apoptosis proteins are related to their subcellular location (Reed and Paternostro, 1999). The subcellular location of apoptosis proteins will not only help us understand the life process and mechanism of programmed cell death but also provide a very important method for understanding the structure and function of proteins (Chou, 2001). It can provide a new perspective for subsequent protein-related tasks such as protein structure prediction and drug-protein relationship prediction (Li et al., 2022a; Li et al., 2022b). However, it is expensive and time-consuming to carry out various experiments to obtain location information (Koroleva et al., 2005). With the explosive growth of protein sequences in the post-genomic era, it is both challenging and necessary to develop an automatic method for quick and accurate prediction of the apoptosis proteins’ subcellular location.

In recent years, serval methods have been proposed for the prediction of apoptosis proteins’ subcellular location. Yu et al. (2012) proposed a prediction method called CELLO, which used multiple SVM classifiers based on N-peptide features. The overall accuracies for their two datasets achieve 87.1% and 90%, respectively. Zhou and Doctor, (2003) established a 98 apoptosis protein data set named ZD98 based on the SWISS-PROT database. They constructed the predictor based on the amino acid composition of the apoptosis protein sequences. The overall success rates of the self-consistent test and jackknife test were 90.8% and 72.5%, respectively. Bulashevska and Eils (2006) used the ZD98 dataset and the jackknife test overall prediction accuracy of the single Bayesian classifier (BC) and hierarchical Bayesian classifier (HensBC) was 85.7% and 89.8% respectively. Chen et al. (2021). proposed a new method to predict the subcellular location of apoptosis proteins by combining dipeptide composition and a discrete increment (ID) algorithm. They predicted the subcellular location of apoptosis proteins based on the main sequence of proteins and the measurement and increase of diversity. According to the latest SWISS-PROT database, they selected 317 apoptosis proteins to establish a data set CL317 and classified them into six subcellular locations (Chen and Li, 2007a). Subsequently, the self-consistent test and jackknife test were conducted, and the overall prediction success rates were 92.1% and 82.7%, respectively. At the same time, they applied this method to ZD98. The overall prediction success rates of the self-consistent test and jackknife test were 94.9% and 90.8%, respectively. Chen and Li, (2007) applied Discrete Incremental Fusion to the dataset. The overall prediction accuracy obtained by the Jackknife test reached 90.8%. For other classes with small samples, the sensitivity reached 91.7%. Later, they combined the ID with a support vector machine (SVM) to propose a new algorithm. For the database of 317 apoptosis proteins in six categories, the overall accuracy of the jackknife test was improved to 85.8%. Zhang et al. (2006) built a larger data set named ZW225. They adopted the feature extraction method based on grouping weight coding, and the overall prediction success rates of self-consistent and jackknife tests were 97.3% and 75.1% respectively. Then they combined the support vector machine with the encoding based on grouped weights feature extraction method, and the overall accuracy of the jackknife test rose to 83.1%.

In this article, we proposed a novel algorithm for apoptosis proteins’ subcellular location prediction. The amphiphilic pseudo amino acid components were used to extract the features from protein sequences. Then, the optimal features were inputted into a machine-learning method to train, test and build a model. The developed approach will be useful for studying apoptosis proteins’ localization and distribution.

## 2 Materials and methods

### 2.1 Datasets

Reliable data is the basis of model construction (Su et al., 2021). Three datasets extracted from the Uniprot (https://www.uniprot.org/) were used to construct the benchmark dataset. The dataset CL317 provided by Chen and Li (2007) consists of 317 apoptosis proteins divided into six subcellular locations with 112 cytoplasmic proteins (*Cyto*), 55 plasma membrane-bound proteins (*Memb*), 52 nuclear proteins (*Nucl*), 47 endoplasmic reticulum proteins (*Endo*), 34 mitochondrial proteins (*Mito*) and 17 secreted proteins (*Secr*). All the accession numbers can be found in the literature (Zhou and Doctor, 2003; Chen and Li, 2007; Zhang et al., 2006). ZW225 is a larger dataset provided by Zhang et al. (2006). It contains 225 apoptosis proteins divided into four subcellular locations of which 41 are *Nucl*, 70 *Cyto*, 25 *Mito* and 89 *Memb*. The dataset ZD98 was generated by Zhou and Doctor, 2003. The 98 apoptosis proteins were classified into four location categories, of which 43 are *Cyto*, 30 *Memb*, 13 *Mito* and 12 other proteins (*Other*). In this study, the jackknife test was applied to build the prediction model and examine the effectiveness of these three datasets.

### 2.2 Feature encoding

We need to convert sequences into vectors in mathematical representation (Amanatidou, and Dedoussis, 2021; Dao et al., 2022a; Jeon et al., 2022; Li H et al., 2022; Nidhi et al., 2022; Sun et al., 2022; Tran and Nguyen, 2022; Wang et al., 2022; Yang et al., 2022; Zhang H et al., 2022). The amino acid composition (ACC) of the protein has a great impact on its subcellular location (Chou and Elrod, 1999a; Awais et al., 2021; Chou and Elrod, 1999b; Rout et al., 2022; Naseer et al., 2021; Manavalan and Patra, 2022; Shoombuatong et al., 2022). By using the ACC to extract features of the protein sequences. a protein sequence can be represented as a 20-D (dimension) vector as follows:

$$P_{k}^{\xi }={\left[p_{k,1}^{\xi },p_{k,2}^{\xi },\ldots ,p_{k,i}^{\xi },\ldots ,p_{k,20}^{\xi }\right]}^{T},\left(i=1,2,\ldots ,20;\;\xi =1,2,\ldots ,\mu ;\;k=1,2,\ldots ,m\right)$$ (1)

In Eq. 1, ξ represents the different subcellular locations of proteins, μ is the total number of subcellular location categories, *k* represents the sequence number in the subcellular position ξ , m is the total number of sequences contained in the subcellular position ξ , and *T* means that the feature vector is expressed in the form of a column vector. $p_{k,i}^{\xi }$ means the occurrence frequency of the amino acid i of the protein sequence *k* in the subcellular position ξ . The amphiphilic pseudo amino acid composition (APAAC) was originally proposed by Chou (2005) to reflect the sequence-order effects by using the hydrophobicity and hydrophilicity of the constituent amino acids in a protein (Hosen et al., 2022; Qian et al., 2022). By using APAAC, a protein sample can be represented as follows:

$${P=\left[p_{1},\ldots ,p_{20},p_{20+1},\ldots ,p_{20+\lambda },p_{20+\lambda +1},\ldots ,p_{20+2\lambda }\right]}^{T}$$ (2)

where the first 20 numbers in Eq. 2 are the classic AAC features, and the next 2λ discrete numbers are sequence-correlation factors, which can be calculated according to the literature (Chou, 2005). For different problems, the optimal value of λ is variable. In this study, the optimal value of λ was selected as the one that yielded the highest overall accuracy through the jackknife test. The APAAC features were generated by the iLearnPlus (Chen, 2021) web server (https://ilearnplus.erc.monash.edu/).

### 2.3 Support vector machine

Support vector machine (SVM) is a powerful supervised machine learning method based on statistical learning theory (Manavalan et al., 2019a). It was originally designed for solving binary classification problems. The basic idea of the generalized linear classifier is as follows: 1) mapping input vector to feature space (possibly high-dimensional space); 2) In the mapped feature space, a separating hyperplane is constructed to separate the two categories (Vapnik, 2019). To sidestep the expensive calculations, the mapping function only involves the relatively low dimensional vector in the input space and the dot product in the feature space. SVM always seeks solutions for global optimization and avoids overfitting. SVM has been successfully applied to many bioinformatic problems (Wei et al., 2017; Wei et al., 2018; Manayalan et al., 2019a; Manayalan et al., 2019b; Ao et al., 2021; Basith et al., 2021; Zeng et al., 2021; Basith et al., 2022; Zhang Q et al., 2022), such as the disease development prediction (Zhang et al., 2020; Zhang et al., 2021a; Ren et al., 2022; Yu et al., 2022), protein prediction (Tang et al., 2018; Tao et al., 2020; Zou et al., 2021; Ao et al., 2022), etc. In this paper, a widely used software LIBSVM (http://www.csie.ntu.edu.tw/∼cjlin/libsvm) (Chang and Lin, 2011) was used to implement the support vector machine. The radial basis function which is defined as $K\left(x_{i},x_{j}\right)=\exp\left(-\gamma {\left\|x_{i}-x_{j}\right\|}^{2}\right)$ was chosen as the kernel function. The regularization parameter *C* and the kernel width parameter *γ* were optimized on the training set using a grid search strategy.

### 2.4 Evaluation methods

At present, there are three main test methods to evaluate the prediction results: the re-substitution test, the Jackknife test and the k-fold cross-validation test (Zhang et al., 2020; Zhang et al., 2021b; Deng et al., 2021; Liu et al., 2021; Tabaie et al., 2021; Ao et al., 2022a; Dai et al., 2022; Dao et al., 2022; Jin et al., 2022; Wei et al., 2022; Xiao et al., 2022; Zhou et al., 2022). Chou and Zhang have discussed in depth the classification performance estimation in bioinformatics and found the Jackknife test and k-fold cross-validation test have extrapolation ability in statistics (Malik et al., 2021; Hasan et al., 2022). In this article, we used the Jackknife test to evaluate the prediction results. The sensitivity (*S*_*n*_), specificity (*S*_*p*_), overall prediction accuracy (*OA*) and Matthew’s correlation coefficient (*MCC*) were used to evaluate the prediction performance of the algorithm (Jiang et al., 2013; Guo et al., 2020; Lv et al., 2020; Xu et al., 2021; Yang et al., 2021; Yu et al., 2021; Han et al., 2022; Zhang Z Y et al., 2022), which are defined as follows:

$$Sn=\frac{TP}{TP+FN}$$ (3)

$$Sp=\frac{TN}{TN+FP}$$ (4)

$$MCC=\frac{TP\times TN-FP\times FN}{\sqrt{\left(TP+FN\right)\left(TP+FP\right)\left(TN+FP\right)\left(TN+FN\right)}}$$ (5)

$$OA=\frac{TP+TN}{TP+TN+FN+FP}$$ (6)

where *TP* represents the number of the positive sample correctly identified, *FN* represents the positive sample wrongly identified as a negative sample, *FP* represents the negative sample wrongly identified as a positive sample, and *TN* represents the negative sample correctly identified (Jia et al., 2020; Li et al., 2021).

## 3 Results and discussion

### 3.1 Model performance

The proposed algorithm based on APACC and SVM was named APACC_SVM. APAAC was generated by the iLearnPlus, with two parameters to be determined, λ and ω namely. In order to obtain ideal results, the selected values of ω were 0.05, 0.1, 0.2, 0.3, 0.4 and 0.5. The selected values of λ were the integers from 2 to 9. The jackknife test was applied to examine APAAC_SVM model. The predictive results for the three apoptosis protein datasets were enumerated in Table 1. When ω = 0.1 and λ = 7, the overall prediction effect was the best for the CL317 dataset. For CL317, the predictive results showed that the overall accuracy was 90.5% in the jackknife test. We noticed the prediction result on the *Secr* was far lower than the other which may be due to the small subset (17 proteins). To improve the accuracy of prediction, it is necessary to collect enough proteins in the dataset.

**TABLE 1:** The predictive results of the three datasets.

Dataset	Location	*Sn*	*Sp*	*MCC*	*OA (%)*
CL317	*Cyto*	0.94	0.91	0.88	90.5
	*Memb*	0.89	0.96	0.91	
	*Mito*	0.88	0.81	0.83	
	*Secr*	0.76	0.76	0.75	
	*Endo*	0.89	0.98	0.92	
	*Nucl*	0.92	0.91	0.90	
ZW225	*Cyto*	0.83	0.82	0.74	84.0
	*Memb*	0.93	0.91	0.87	
	*Mito*	0.68	0.85	0.73	
	*Nucl*	0.76	0.72	0.68	
ZD98	*Cyto*	0.95	0.98	0.94	93.9
	*Memb*	0.97	0.94	0.93	
	*Mito*	0.92	0.92	0.91	
	*Other*	0.83	0.91	0.85	

When ω = 0.3 and λ = 7, the overall prediction effect was the best for the ZW225 dataset. For ZW225, the jackknife test showed the overall accuracy was 84.0%. According to the prediction results obtained from the training of the ZW225 dataset, although the prediction effect was not as good as CL317, the overall appearance was similar. In the subsets *Mito* and *Nucl* (25 and 41 proteins, respectively) with fewer sequences, the prediction accuracies were significantly lower than the others. It showed that expanding the data scale was important for prediction improvement.

When ω = 0.2 and λ = 7, the overall prediction effect was the best for the ZD98 dataset. The predictive results for ZD98 apoptosis protein sets showed that the overall accuracy was 93.9% in the jackknife test.

### 3.2 Model comparison

To prove the prediction ability of our APAAC_SVM algorithm, we compared our model with previous algorithms. For the CL317 dataset, Chen and Li proposed the ID method and ID-SVM method, Zhang Li et al. used the DF-SVM method for the apoptosis proteins’ subcellular location prediction, respectively. The comparison results were shown in Table 2. It can be seen from the table that our APAAC-SVM method significantly improved the prediction results in both the overall prediction accuracy and in each subcellular location, especially in *Cyto*, *Mito* and *Endo*.

**TABLE 2:** Comparison of prediction performance for different methods on the CL317 dataset.

Localization	ID	ID-SVM	DF-SVM	APAAC-SVM
	*Sn* (%)	*Sn* (%)	*Sn* (%)	*Sn* (%)
*Cyto*	81.3	91.1	92.9	93.8
*Memb*	81.8	89.1	85.5	89.1
*Mito*	85.3	79.4	76.5	88.2
*Secr*	88.2	58.8	76.5	76.5
*Nucl*	82.7	73.1	93.6	92.3
*Endo*	83.0	87.2	86.5	89.4
*OA* (%)	82.7	84.2	88.0	90.5

For the ZW225 dataset, Zhang and Wang used the EBGW-SVM and DF-SVM methods, and Chen and Li used the ID-SVM method for prediction. The prediction model performances were shown in Table 3. It can be seen from Table 3 that the overall prediction accuracy of each method was relatively close. However, the APAAC-SVM algorithm achieved good prediction accuracy in both the *Memb* and *Nucl*. It indicated that our algorithm was relatively ideal.

**TABLE 3:** Comparison of prediction performance for different methods on the ZW225 dataset.

Localization	EBGW-SVM	DF-SVM	ID-SVM	APAAC-SVM
	Sn (%)	Sn (%)	*Sn* (%)	*Sn* (%)
*Cyto*	90.0	87.1	92.9	82.9
*Memb*	93.3	92.1	91.0	93.3
*Mito*	60.0	64.0	69.0	68.0
*Nucl*	63.4	73.2	73.2	75.6
*OA* (%)	83.1	84.0	85.8	84.0

For the ZD98 dataset, Zhou and Doctor, Huang Jing, Bulashevska, Eils, Chen and Li have all conducted research. They have respectively applied covariant discrimination algorithm, SVM algorithm, Bayesian discrimination algorithm and discrete incremental fusion algorithm. The predicted results were shown in Table 4. The overall prediction accuracy of the APAAC-SVM method was 93.9% for the ZD98 dataset, which was higher than other methods. When the Jackknife test was used, the overall prediction accuracy was improved by 21.3% compared with the covariant discriminant algorithm of Zhou and Doctor. Compared with the Bayesian discriminant method of Bulashevska Eils, the overall prediction accuracy was increased by about 8.1%. For a small sample of other apoptosis proteins in the data set, the sensitivity of these two methods was only 25% and 50%, while the sensitivity of this method can reach 83.33%. Compared with Huang Jing’s SVM algorithm, this method had a higher overall prediction success rate, which was increased by about 3%; Moreover, the sensitivity of *Cyto* was higher, which reached 95.3%. Compared with the discrete incremental fusion method of Chen Yingli and Li Qianzhong, the overall prediction success rate of this method was also higher.

**TABLE 4:** Comparison of prediction performance for different methods on the ZD98 dataset.

	Covariant	SVM	BC		Hensbc		IDF			APAAC-SVM		
	*Sn*(%)	*Sn*(%)	*Sn*(%)	*MCC*	*Sn*(%)	*MCC*	*Sn*(%)	*Sp*(%)	*MCC*	*Sn*(%)	*Sp*(%)	MCC
*Cyto*	97.7	86.0	90.7	0.81	95.3	0.89	90.7	95.1	0.87	95.3	97.6	0.94
*Memb*	73.3	90.0	90.0	0.83	90.0	0.83	90.0	93.1	0.88	96.7	93.5	0.93
*Mito*	30.8	100	92.3	0.83	92.3	0.83	92.3	70.6	0.77	92.3	92.3	0.91
*Other*	25.0	100	50.0	0.57	66.7	0.80	91.7	100	0.95	83.3	90.9	0.85
*OA* (%)	72.5	90.8	85.7	—	89.8	—	90.8	—	—	93.9	—	—

By compared with previous studies, it can be found that the APAAC-SVM method was better for category prediction with more sequence data. It showed that this method was more suitable for the prediction of apoptosis protein subcellular locations in the case of increasing sequence data, and it also had an optimistic application prospect in future research.

## 4 Conclusion

Previous apoptosis proteins’ subcellular location analysis demonstrated that information in protein sequence has a great influence on its subcellular localization. However, the performance of the proposed algorithms for apoptosis proteins’ subcellular location prediction is inadequate. This study selected three apoptosis protein sequence datasets CL317, ZD98 and ZW225 to develop a new prediction algorithm. The APAAC feature extraction method and SVM were combined to predict the subcellular location of apoptosis proteins. Through the reasonable selection of parameters, our algorithm APAAC_SVM achieved jackknife test prediction accuracy of 90.5%, 93.9% and 84.0% on CL317, ZD98 and ZW225, respectively. Compared with other methods, APAAC-SVM improved the prediction performance.

## Data Availability

The original contributions presented in the study are included in the article/supplementary material, further inquiries can be directed to the corresponding authors.
